# Supplementary material for: Tunable transmission of quantum Hall edge channels with full degeneracy lifting in split-gated graphene devices
Source: Nat Commun. 2017 Apr 13;8:14983. doi: 10.1038/ncomms14983 (PMC5399284; doi:10.1038/ncomms14983)
Supplement: Supplementary Information — Supplementary Figures, Supplementary Tables, Supplementary Notes and Supplementary References [file ncomms14983-s1.pdf]

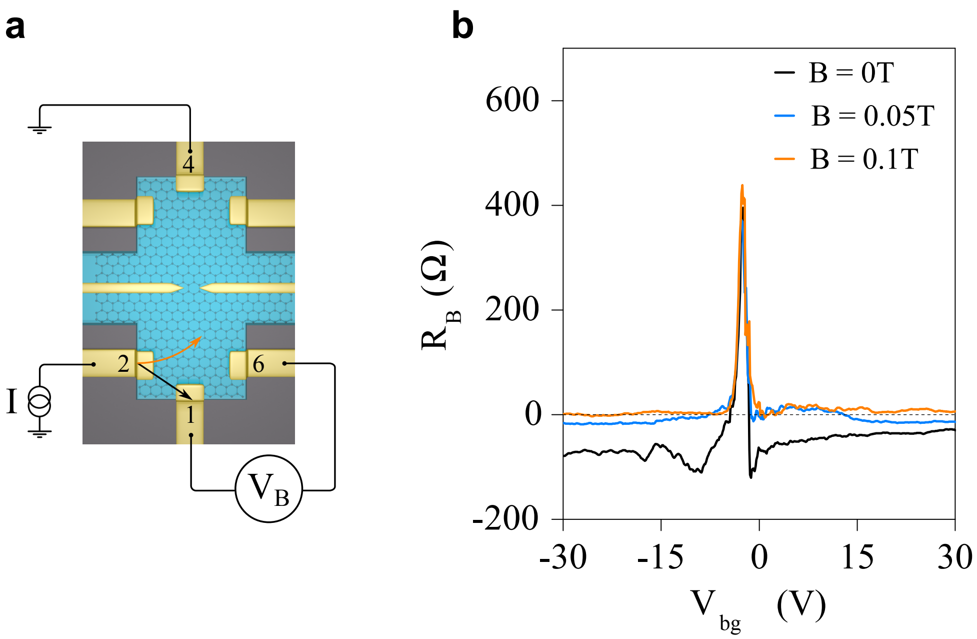

Supplementary Figure 1: **Evidence for ballistic transport in the bend resistance** (**a**) Schematic of the measurement configuration for non local, bend resistance. Electron trajectories are shown by arrows at zero (black) and increasing (red) magnetic field. (**b**) The bend resistance $R_B = R_{24,16}$ as a function of the back-gate V_{bg} becomes negative beyond the Dirac point at zero magnetic fields. With increasing magnetic field, the electronic trajectory is curved preventing electrons from arriving at the contacts 1 and 6, leading to suppression of the negative component.

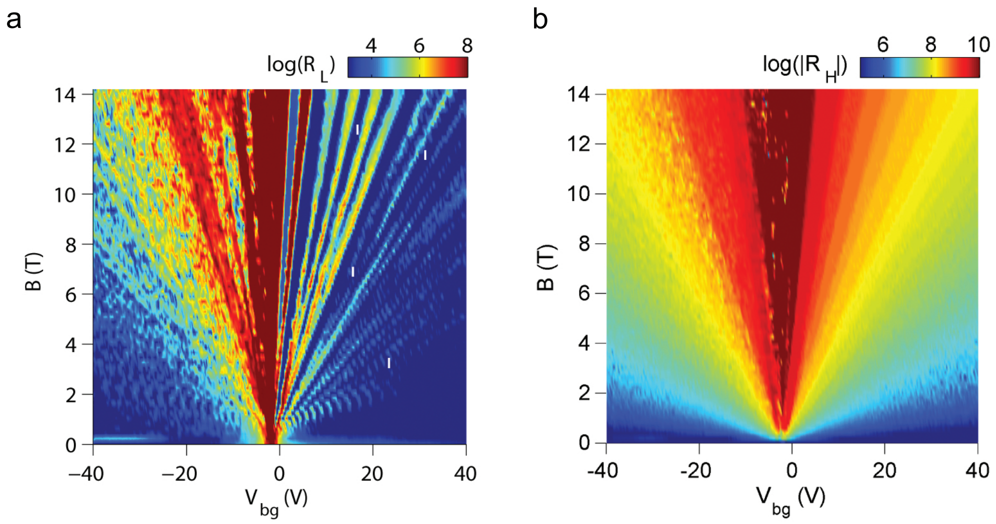

Supplementary Figure 2: **Landau level fan diagram for electrons and holes.** (**a**) Landau level fan diagram displaying $log(R_L)$ versus B and V_{bg}. (**b**) colormap of $log(|R_H|)$ versus B and V_{bg}. Those are the same data as in Fig 1d of main text.

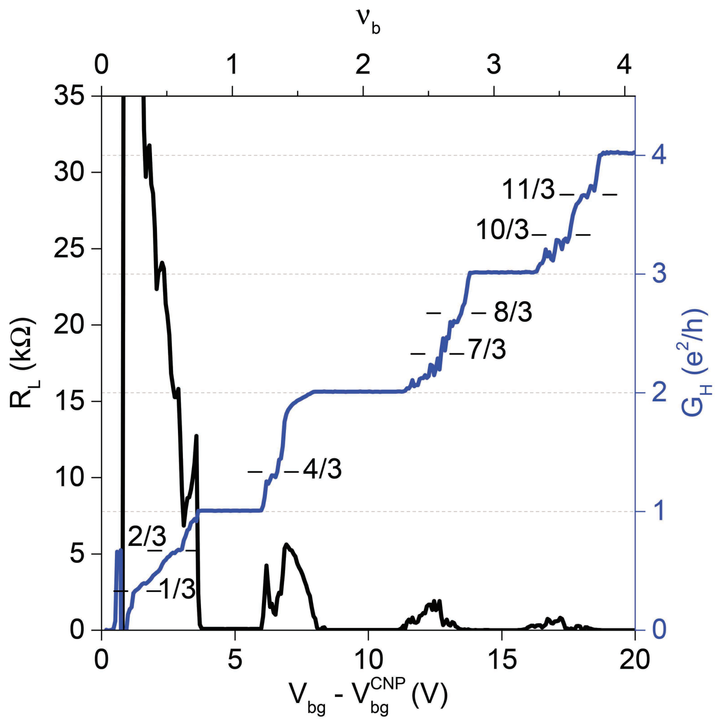

Supplementary Figure 3: **Fractional QH states.** Transverse Hall conductance G_H and longitudinal resistance R_L versus $V_{bg} - V_{bg}^{CNP}$ measured at $0.05\,$K and $14\,$T. Top axis is the corresponding bulk filling factor ν_b.

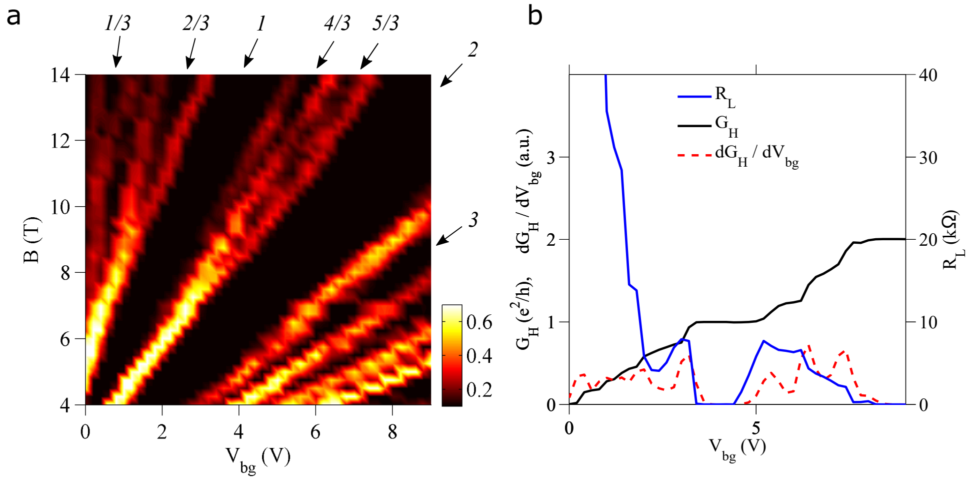

Supplementary Figure 4: **Landau fan diagram for fractional QH states.** (**a**) Colormap of the derivative of the Hall conductance dG_H/dV_{bg} plotted versus B and V_{bg} ($T = 0.05$K). Labels of the black arrows indicate the filling factor. (**b**) Plot of G_H, R_L and dG_H/dV_{bg} (linecut of (**a**)) versus V_{bg} measured at 14T.

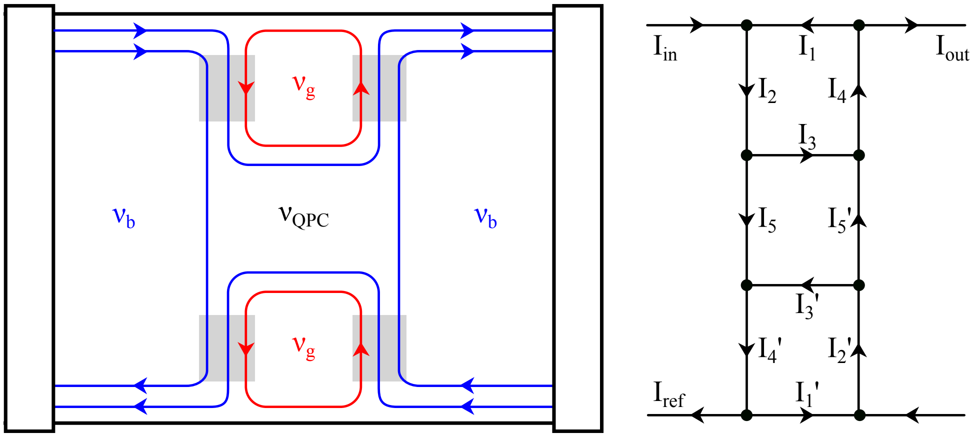

Supplementary Figure 5: **Edge channel configuration for** $\nu_{QPC} \geq 0$**.** Schematic drawing of the electon (blue) and hole (red) edge channel configuration in the QPC and the electric circuit for calculating the outgoing current I_{out}. The grey areas indicate the p-n interfaces where edge channels equilibrate.

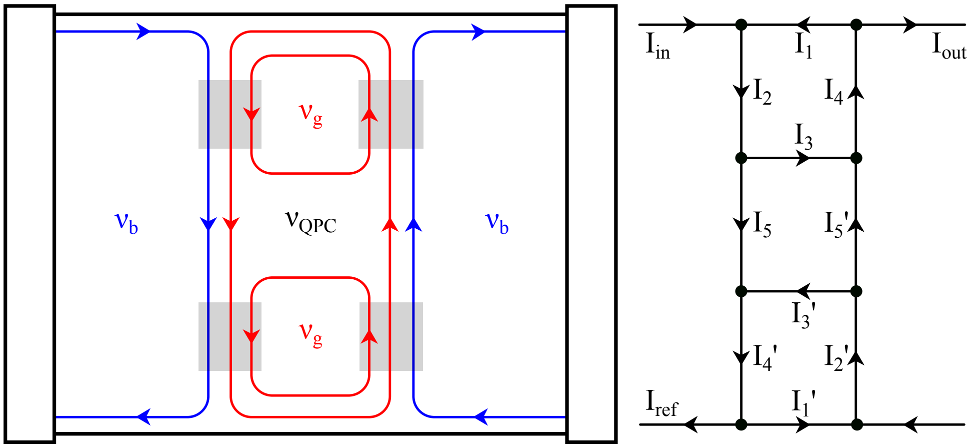

Supplementary Figure 6: **Edge channel configuration for** $\nu_{QPC} < 0$**.** Schematic drawing of the electon (blue) and hole (red) edge channel configuration in the QPC and the electric circuit for calculating the outgoing current I_{out}. The grey areas indicate the p-n interfaces where edge channels equilibrate.

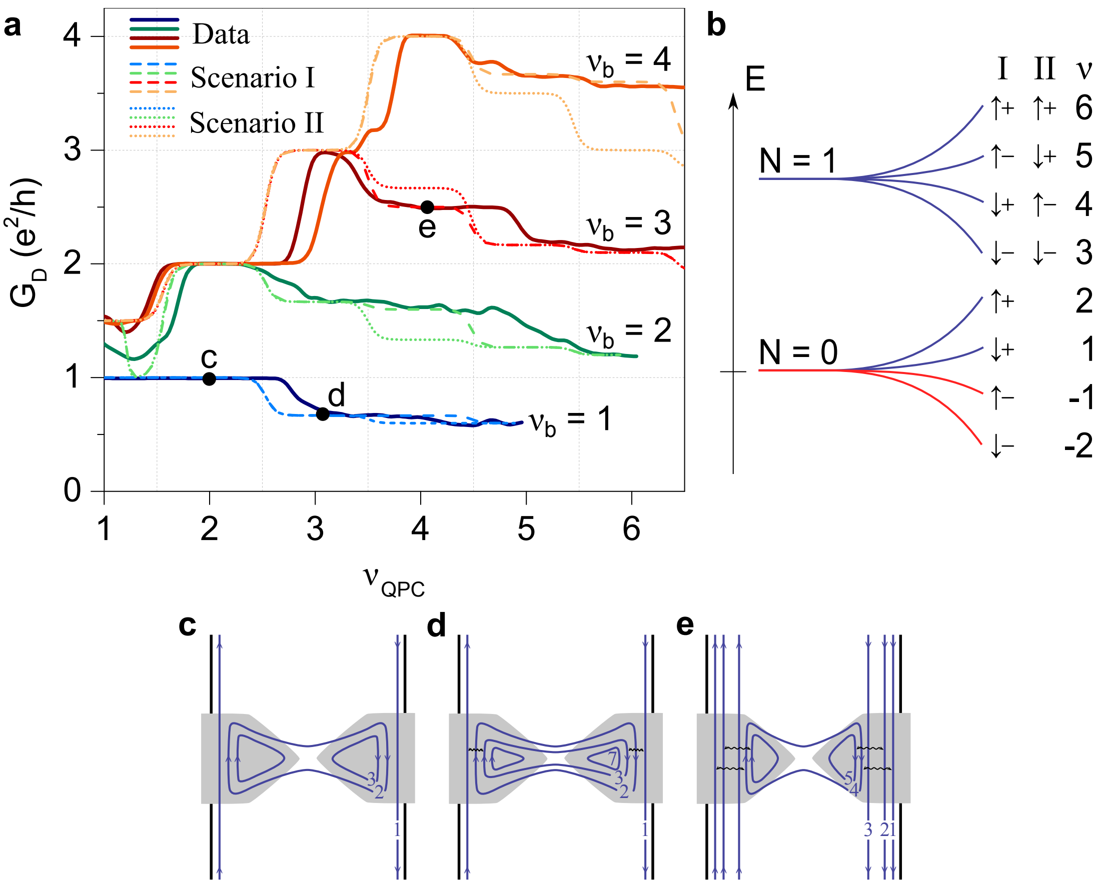

Supplementary Figure 7: **Selective equilibration in the unipolar regime** (**a**) Results on the diagonal conductance extracted from the conductance map for $V_{sg} > 0$ at various bulk filling factors. The theoretical curves are calculated from (16) which includes that equilibration only occurs between states of identical spin polarisations. The dashed theoretical curves are based on scenario I and the dotted curves represent the solution of scenario II. (**b**) Energy diagram of the broken symmetry states of the $N = 0$ and $N = 1$ Landau levels. The two scenarios (I, II) indicate the two orders of the splitting of the degeneracies of the $N = 1$ Landau level. (**c-e**) Schematics of edge channel configurations at the black dots labeled c-e in (a). (**c**) $(\nu_b, \nu_{QPC}) = (1, 2)$, (**d**) $(1, 3)$, (**e**) $(3, 4)$

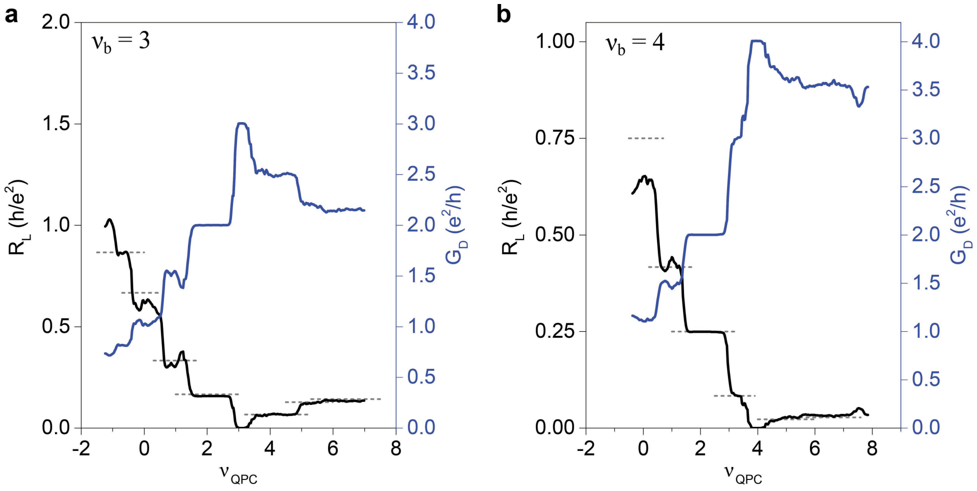

Supplementary Figure 8: **Longitudinal resistance plateaux.** Longitudinal resistance R_L and diagonal conductance G_D versus ν_{QPC} at a bulk filling factor of (a) $\nu_b = 3$ and (b) $\nu_b = 4$. Expected resistance plateaux are indiciated with dotted lines, showing good agreement with the data.

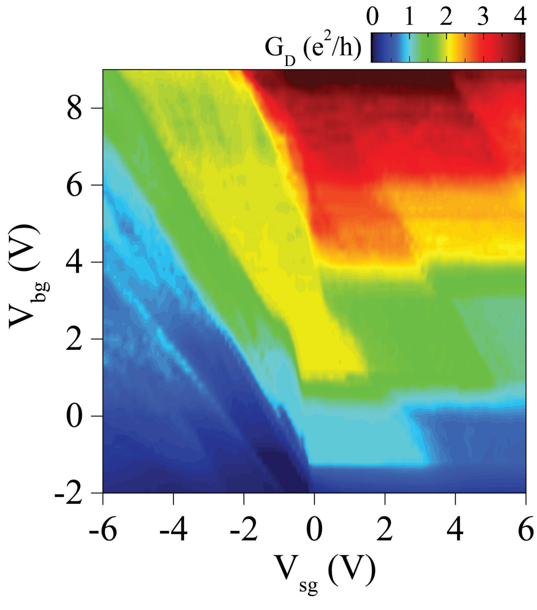

Supplementary Figure 9: **Diagonal conductance map of a second QPC device.** G_D versus V_{bg} and V_{sg} measured at 7T and 0.05K.

Supplementary Table 1: Conductance plateaux G_D for $V_{sg} > 0$. The errors are obtained from the minimal and maximal value of each plateau. Conductance values G calculated with eq. (16) for scenario I and II.

ν_b	ν_{QPC}	G_D measured $\left(\frac{e^2}{h}\right)$	G (I) $\left(\frac{e^2}{h}\right)$	G (II) $\left(\frac{e^2}{h}\right)$
1	2	0.995 ± 0.005	1	1
	3	0.70 ± 0.03	$\frac{2}{3} \simeq 0.67$	$\frac{2}{3} \simeq 0.67$
	4	0.63 ± 0.05	$\frac{3}{5} = 0.60$	$\frac{2}{3} \simeq 0.67$
2	3	1.67 ± 0.03	$\frac{5}{3} \simeq 1.67$	$\frac{5}{3} \simeq 1.67$
	4	1.62 ± 0.02	$\frac{8}{5} = 1.60$	$\frac{4}{3} \simeq 1.33$
	5	1.45 ± 0.10	$\frac{19}{15} \simeq 1.27$	$\frac{19}{15} \simeq 1.27$
3	4	2.49 ± 0.03	$\frac{5}{2} = 2.50$	$\frac{8}{3} \simeq 2.67$
	5	2.20 ± 0.02	$\frac{13}{6} \simeq 2.17$	$\frac{13}{6} \simeq 2.17$
	6	2.12 ± 0.02	$\frac{21}{10} = 2.10$	$\frac{21}{10} = 2.10$
4	5	3.65 ± 0.01	$\frac{11}{3} \simeq 3.67$	$\frac{7}{2} = 3.50$
	6	3.56 ± 0.02	$\frac{18}{5} = 3.60$	3

Supplementary Note 1: Sample fabrication

We used the van der Waals transfer technique to encapsulate graphene in between hBN following the method shown in [1]. Both hBN and graphene flakes were separately exfoliated onto SiO_2/Si(p++) substrates. Atomically flat and clean hBN flakes and clean graphene flakes were preselected by means of optical search followed by atomic force microscopy imaging. A stamp of polydimethylsiloxane (PDMS) covered by a polypropylene carbonate (PPC) layer was used for the successive van der Waals pick-up and stacking of the flakes. The resulting heterostructures of hBN / graphene / hBN were imaged by AFM to identify graphene location and reject the region affected by bubble and blisters that can affect transport properties. For the fabrication of QPC devices, the heterostructures were fully etched in a CHF_3 + O_2 plasma with a polymethyl methacrylate/hydrogen silsesquioxane (PMMA/HSQ) mask patterned in such a way that some edges of the graphene remain encapsulated. Such edges are drawn in white dotted lines in Fig. 1b. This patterned etching enables to design in a single metal deposition step both one-dimensional contacts on the graphene etched edges, and the split-gates passing on the edges of the heterostructure where graphene is not accessible, avoiding any short or use of additional gate dielectric layers.

Supplementary Note 2: Hole doping

In the main text, we focused our analysis of the data on the electron side because we obtained more resistive contacts on the hole side, leading to not well defined Landau level fan and Hall quantization for hole doping (see Supplementary Figure 2). Such an electron-hole asymmetry usually stems from a charge transfer from the metallic contact to the graphene. In our devices, contacts induce a graphene electron-doped region in their vicinity, thus forming a p-n junction when the bulk is hole-doped. In the quantum Hall regime, in line with the mitigated equili-

bration observed in the study of the QPC, the contact transmission is reduced due to these p-n junctions, resulting in a bad quantization and a noisy longitudinal resistance oscillations (See Supplementary Figure 2).

Supplementary Note 3: Negative non-local resistance

Non-local resistance $R_B = R_{24,16}$ (also referred to as bend resistance), is measured by applying current between contacts 2 and 4 while voltage is measured between contacts 1 and 6 (Fig. 1a). This resistance as a function of backgate voltage is presented in Supplementary Figure 1b for $B = 0, 0.05$ and $0.1\,\mathrm{T}$. At zero magnetic field, R_B is negative away from the charge neutrality point. With increasing magnetic field, R_B increases, as electron trajectories are bent due to the Lorentz force and are no longer incident on the voltage probe (contact 1), indicating ballistic transport [2, 1].

Supplementary Note 4: Fractional quantum Hall regime

At our base temperature of $0.05\,\mathrm{K}$ and magnetic field of $14\,\mathrm{T}$, we observe fractional quantum Hall plateaux in the transverse Hall conductance G_H at filling factors $\nu_b = 1/3$, $2/3$, $4/3$, $8/3$, $10/3$ and $11/3$, which are accompanied by minima in the longitudinal resistance R_L (see Supplementary Figure 3) [3, 4, 5, 6]. The Hall conductance is calculated by $G_H = \frac{R_H}{R_H^2 + \left(\frac{W}{L} R_L\right)^2}$, where W is the width and L the length of the device. The $7/3$ state is not clearly visible in this line-cut but is revealed by pinching off the QPC (see main text). Note that the $5/3$ state is not resolved in this set of data, as reported in previous works [5, 6].

Fractional quantum Hall plateaux are also resolved in a second QPC device (see Supplementary Note). Supplementary Figure 4a displays the derivative dG_H/dV_{bg} versus B and V_{bg}. Minima in dark color indicate plateaux in G_H. Beside the large minima at integer filling factors 1 and 2, other minima disperse in the $B - V_{bg}$ plane at fractional filling factors $\nu_b = 1/3$,

$2/3$, $4/3$, and $5/3$, and coincide with minima in the longitundial resistance (see Supplementary Figure 4b). Supplementary Figure 4a thus provides the Landau fan diagram for the fractional quantum Hall states observed in our samples.

Supplementary Note 5: Equilibration for the QPC geometry in graphene

As described in the main text, the expression for diagonal conductance of a pnp-junction with two filling factors [7, 8, 9, 10] is no longer applicable to the QPC geometry which involves the three filling factors ν_b, ν_g and ν_{QPC}. We derive in this section the two-terminal conductance for the regions I and III for such a QPC geometry – the two-terminal conductance is equal to the four-terminal diagonal conductance G_D measured in our experiment– considering spin selective equilibration [11] between electron bulk edge channels and hole edge channels localized underneath the split-gates. Conductance in region II is described by Supplementary Equation (1) in the main text.

Region I - Conductance in the bipolar regime

Equilibration in region I involves two cases that depend on the sign of ν_{QPC}. When $\nu_{QPC} < 0$ localized hole states underneath the split-gates extend across the QPC, whereas for $\nu_{QPC} \geq 0$ electron edge channels from the bulk pass through the QPC and the hole states remain localized beneath each split-gate. We derive below the diagonal conductance for both cases.

Configuration $\nu_{QPC} \geq 0$

In this configuration electron edge channels are transmitted through the QPC while hole states are localized underneath the split-gates. Supplementary Figure 5 sketches the edge channels of the bulk and those induced by the split-gate for the QPC geometry. The incoming current I_{in}

splits into two branches in the vicinity of the QPC. One part of the current is transmitted through the constriction of filling factor $\nu_{QPC} \geq 0$ and the rest is backscattered along the bulk/split-gate interface.

We consider four segments of pn interfaces where electron bulk edge channels equilibriate with hole-doped edge channels. There are highlighted with grey areas in Supplementary Figure 5. Assuming that the incoming current impinging each pn interface is equally distributed among the co-propagating electron and hole channels, we can write:

$$I_1 = rI_4 \tag{1}$$

$$I'_1 = rI'_4 \tag{2}$$

$$I_3 = r'I_2 \tag{3}$$

$$I'_3 = r'I'_2 \tag{4}$$

with $r = \frac{|\nu_g|}{|\nu_b|+|\nu_g|}$ and $r' = \frac{|\nu_{QPC}|+|\nu_g|}{|\nu_b|+|\nu_g|}$. Current conservation at the eight nodes leads to:

$$I_2 = I_1 + I_{in} = I_3 + I_5 \tag{5}$$

$$I_4 = I_1 + I_{out} = I_3 + I'_5 \tag{6}$$

$$I'_2 = I'_1 = I'_3 + I'_5 \tag{7}$$

$$I'_4 = I'_1 + I_{ref} = I'_3 + I_5 \tag{8}$$

$$\tag{9}$$

in which we assume that no current is injected from the right lead. The outgoing current I_{out} as a function of I_{in} reads:

$$I_{out} = \frac{r - 2rr' + r'}{1 + r - 2rr'} I_{in} \tag{10}$$

The incoming current I_{in} is given by the number of incoming edge channels ν_b and the chemical potential difference $\mu_1 - \mu_2$ between the two leads: $I_{in} = \frac{e^2}{h}|\nu_b|\,(\mu_1 - \mu_2)$. Together with

$G = \frac{I_{out}}{\mu_1 - \mu_2}$, we obtain the conductance

$$G^{\nu_b,\nu_g,\nu_{QPC}} = \frac{e^2}{h}|\nu_b|\frac{2|\nu_b|\,|\nu_g| + |\nu_{QPC}|(|\nu_b| - |\nu_g|)}{3|\nu_b|\,|\nu_g| + |\nu_b|^2 - 2|\nu_{QPC}|\,|\nu_g|} \tag{11}$$

which depends solely on the three filling factors ν_b, ν_g and ν_{QPC}.

Configuration $\nu_{QPC} < 0$

In the second configuration when $\nu_{QPC} < 0$, the hole states from underneath the split-gates extend across the QPC (see Supplementary Figure 6). Both hole states propagating across the QPC and those localized underneath the split-gates can equilibrate with the back-reflected electron edge channels of the bulk. As for the previous configuration equilibration occurs at the same four pn-interfaces shown in grey in Supplementary Figure 6. Similar calculations lead to :

$$G^{\nu_b,\nu_g,\nu_{QPC}} = \frac{e^2}{h}|\nu_b|\frac{2|\nu_b|\,|\nu_g| - |\nu_{QPC}|(|\nu_b| - |\nu_g|)}{3|\nu_b|\,|\nu_g| + |\nu_b|^2 + 2|\nu_{QPC}|\,|\nu_g|} \tag{12}$$

Supplementary Equations (11) and (12) are identical apart from three sign changes given by the sign change of ν_{QPC}. They can thus be summarized into a single equation describing both configurations:

$$G^{\nu_b,\nu_g,\nu_{QPC}} = \frac{e^2}{h}|\nu_b|\frac{2|\nu_b|\,|\nu_g| + \nu_{QPC}(|\nu_b| - |\nu_g|)}{3|\nu_b|\,|\nu_g| + |\nu_b|^2 - 2\nu_{QPC}\,|\nu_g|} \tag{13}$$

In order to include spin selection, we extend Supplementary Equation (13) by summing over the spin:

$$G = \sum_{\sigma=\uparrow,\downarrow} G^{\nu_b^\sigma,\nu_g^\sigma,\nu_{QPC}^\sigma} \tag{14}$$

$$G^{\nu_b^\sigma,\nu_g^\sigma,\nu_{QPC}^\sigma} = \frac{e^2}{h}|\nu_b^\sigma|\frac{2|\nu_b^\sigma||\nu_g^\sigma| + \nu_{QPC}^\sigma(|\nu_b^\sigma| - |\nu_g^\sigma|)}{3|\nu_b^\sigma||\nu_g^\sigma| + |\nu_b^\sigma|^2 - 2\,\nu_{QPC}^\sigma|\nu_g^\sigma|} \tag{15}$$

In (15), ν_b^σ, ν_g^σ and ν_{QPC}^σ count the number of edge channels of identical spins involved in the equilibration.

Region III - Spin-selective equilibration for $V_{sg} > 0$

For $V_{sg} > 0$, the charge carrier density is larger underneath the split-gates than in the bulk ($\nu_g > \nu_b$). Thus all electron bulk edge channels pass underneath the split-gates along the edges of the graphene flake. The additional $\nu_g - \nu_b$ electron edge channels are either localized underneath the split-gates or extend across the QPC (see configuration in Fig. 2i). Equilibration only has an impact on the conductance if the edge channels underneath the split-gates extend across the QPC, connecting the counter-propagating bulk edge channels and introducing backscattering. The number of such localized edge channels connecting the counter-propagating bulk edge channels is given by $\nu_{QPC} - \nu_b$. As a result, the device becomes equivalent to an nn'n-junction. The two-terminal conductance for spin-selective equilibration thus reads [11]:

$$G = \frac{e^2}{h} \sum_{\sigma=\downarrow,\uparrow} \frac{|\nu_b^\sigma|\,|\nu_{QPC}^\sigma|}{2|\nu_{QPC}^\sigma| - |\nu_b^\sigma|} \tag{16}$$

Supplementary Figure7a displays diagonal conductance line-cuts extracted from the conductance map of Fig. 2a as a function of ν_{QPC} for $\nu_b = 1$ to 4. For $\nu_{QPC} > \nu_b$, several conductance plateaux at non-integer values are observed in each curves. As shown by Amet et al. [11], Supplementary Equation (16) enables to discriminate the spin polarization of the sub-Landau levels. We detail below the analysis of the conductance curves at $\nu_b = 1$ and 3.

Case of $\nu_b = 1$

The $\nu_b = 1$ curve in Supplementary Figure 7a has a large plateau of $G_D = \frac{e^2}{h}$ at $\nu_b = \nu_{QPC} = 1$,which corresponds to the configuration where the bulk edge channel passes underneath the split-gates. The conductance remains quantized at $\frac{e^2}{h}$ even for $\nu_{QPC} = 2$ when the second edge channel extends across the QPC (see Supplementary Figure 7c). The fact that the conductance does not decrease indicates that no equilibration occurs between the states $\nu_b = 1$ and $\nu_{QPC} = 2$ due to their opposite spin polarization [11] (see Landau level diagram in Supplementary Figure 7b).

The conductance of the $\nu_b = 1$ curve then decreases to $0.7\frac{e^2}{h}$ at $\nu_{QPC} = 3$ indicating equilibration between the $\nu_b = 1$ state and the $\nu_{QPC} = 3$ state, which share the same spin-up polarization (see Supplementary Figure 7d). Applying Supplementary Equation (16) with $\nu_b^{\uparrow} = 1$ and $\nu_{QPC}^{\uparrow} = 2$ leads to $G_D = G^{\nu_b^{\uparrow},\nu_{QPC}^{\uparrow}} = \frac{2}{3}\frac{e^2}{h}$ in good agreement with the measured plateau. Note that here $G^{\nu_b^{\downarrow},\nu_{QPC}^{\downarrow}} = 0$ as $\nu_b^{\downarrow} = 0$.

Equilibration with higher Landau levels

Spin-selective equilibration depends on the order of the lifting of the spin and valley degeneracies in the $N \geq 1$ Landau levels. There are two possible scenarios. In scenario I, the spin degeneracy lifts first. In scenario II the valley degeneracy lifts first. These two scenarios lead to broken symmetry states with different spin and valley polarisation. The resulting spin-selective equilibration in pnp junctions, therefore, can be used to discriminate between the two scenarios (Supplementary Figure 7b)

We summarize in Supplementary Table 1 the measured values of the diagonal conductance plateaux for $V_{sg} > 0$, together with the expected conductance values given by Supplementary Equation (16) for scenarios I and II. The measured diagonal conductance along with the theoretical diagonal conductance for both scenarios I and II are plotted in Supplementary Figure 7a.

Supplementary Note 6: Longitudinal resistance

Supplementary Figure 8 displays the longitudinal resistance and diagonal conductance, R_L and G_D versus ν_{QPC}, both measured simultaneously at 7T. R_L exhibits plateaux at each integer values of ν_{QPC}.

The expected values of R_L:

$$R_L = \frac{1}{G_D} - R_H = \frac{1}{G_D} - \frac{1}{\nu_b} \tag{17}$$

computed with the above analysis of G_D in the three different regions are indicated by horizontal lines in Supplementary Figure 8. The good agreement with the measured plateaux confirmed the consistency of our measurements.

Supplementary Note 7: Second QPC device

The conductance of a second QPC device is shown in Supplementary Figure 9. We find quantitatively the same results as for the QPC device presented in the main text. For negative V_{sg} the conductance features diagonal strips with the same quantized values of $G_D = e^2/h$ at $\nu_{QPC} = 0$, $G_D = 3/2e^2/h$ at $\nu_{QPC} = 1$, $G_D = 2e^2/h$ at $\nu_{QPC} = 2$... For positive V_{sg}, we observe the same quantization of the rhombi in region III leading to the same conclusion as in previous section.

Supplementary References

[1] Wang, L. *et al.* One-Dimensional Electrical Contact to a Two-Dimensional Material. *Science* **342**, 614–617 (2013).

[2] Mayorov, A. S. *et al.* Micrometer-Scale Ballistic Transport in Encapsulated Graphene at Room Temperature. *Nano Lett.* **11**, 2396–2399 (2011).

[3] Du, X., Skachko, I., Duerr, F., Luican, A, & Andrei, A. Y. Fractional quantum Hall effect and insulating phase of Dirac electrons in graphene. *Nature* **462**, 192–195 (2009).

[4] Bolotin, K.I., Ghahari, F., Shulman, M. D., Stormer, H. L. & Kim, P. Observation of the fractional quantum Hall effect in graphene. *Nature* **462**, 196–199 (2009).

[5] Dean, C. R. *et al.* Multicomponent fractional quantum Hall effect in graphene. *Nature Phys.* **7**, 693–696 (2011).

[6] Amet, F. *et al.* Composite fermions and broken symmetries in graphene. *Nature Commun.* **6**, 5838 (2014).

[7] Abanin, D. A. & Levitov, L. S. Quantized Transport in Graphene p-n Junctions in a Magnetic Field. *Science* **317**, 641–643 (2007).

[8] Özyilmaz, B., and Jarillo-Herrero, P., Efetov, D., Abanin, D. A., Levitov, L. S., & Kim, P. Electronic Transport and Quantum Hall Effect in Bipolar Graphene p-n-p Junctions. *Phys. Rev. Lett.* **99**, 166804 (2007).

[9] Williams, J. R., DiCarlo, L. & Marcus, C. M. Quantum Hall Effect in a Gate-Controlled p-n Junction of Graphene. *Science* **317**, 638–641 (2007).

[10] Ki, D.-K. & Lee, H.-J. Quantum Hall resistances of a multiterminal top-gated graphene device. *Phys. Rev. B* **79**, 195327 (2009).

[11] Amet, F., Williams, J. R., Watanabe, K., Taniguchi, T. & Goldhaber-Gordon, D. Selective Equilibration of Spin-Polarized Quantum Hall Edge States in Graphene. *Phys. Rev. Lett.* **112**, 196601 (2014).
